# Impact of Smokeless Tobacco on Salivary Antioxidant Systems and Oral Health: A Comparative Study Among Individuals With and Without Tobacco Pouch Keratosis

**DOI:** 10.7759/cureus.85229

**Published:** 2025-06-02

**Authors:** Varsha R Patil, Karpagaselvi Sanjai

**Affiliations:** 1 Department of Oral Pathology and Microbiology, Vydehi Institute of Dental Sciences and Research Centre, Bangalore, IND

**Keywords:** frap assay, oral mucosal lesions, oxidative stress, preventive dentistry, salivary antioxidants, salivary flow rate, salivary ph, smokeless tobacco, tobacco-induced oral changes, tobacco pouch keratosis

## Abstract

Background

The widespread use of smokeless tobacco, particularly in India, is a major public health concern, contributing to increased oral cancer cases. Tobacco pouch keratosis, an early sign of oral mucosal damage, is commonly observed in regular smokeless tobacco users. This study examines the impact of smokeless tobacco on salivary antioxidant systems, comparing individuals with and without tobacco pouch keratosis to healthy controls.

Materials and methods

A total of 63 participants were divided into three groups: smokeless tobacco users without oral lesions (group 1, n=23), users with tobacco pouch keratosis (group 2, n=20), and healthy controls (group 3, n=20). Salivary parameters, including flow rate, pH, and total antioxidant capacity (measured by the ferric reducing antioxidant power [FRAP] assay), were analyzed. A descriptive analysis was conducted for both continuous and categorical variables. For inferential statistics, the Mann-Whitney U test was used to compare the duration and frequency of tobacco use. A one-way ANOVA, followed by Tukey's post-hoc test, was applied to compare the mean salivary flow rate and salivary pH levels. Spearman’s correlation test was employed to assess the relationship between total antioxidant levels and various parameters. The level of statistical significance was set at *p* < 0.05.

Results

The mean duration of tobacco use was 6.30 years in group 1 and 18.10 years in group 2 (p=0.001). The mean salivary flow rates were 0.272 ± 0.137 mL/min (group 1), 0.159 ± 0.094 mL/min (group 2), and 0.277 ± 0.192 mL/min (group 3), with a p-value of 0.02. The mean pH levels were 6.999 ± 0.322 (group 1), 6.650 ± 0.355 (group 2), and 6.943 ± 0.360 (group 3), with a *p-*value of 0.004. Group 3 had significantly higher total salivary antioxidant levels (*p*=0.001).

Conclusions

Smokeless tobacco use, particularly in individuals with tobacco pouch keratosis, is associated with reduced salivary flow rate, lower (more acidic) pH, and decreased total antioxidant levels. There is a strong negative correlation between salivary antioxidant levels and the duration and frequency of smokeless tobacco use.

## Introduction

The global prevalence of both smoked and smokeless tobacco presents a major public health challenge [1]. In India, where smokeless tobacco use is widespread, there has been a concerning increase in oral cancer cases, often linked to tobacco consumption [2]. Tobacco pouch keratosis, an early sign of oral mucosal damage, is commonly seen in smokeless tobacco users. Tobacco contains numerous harmful chemicals that induce oxidative stress, leading to the production of free radicals. These free radicals have the potential to damage DNA, proteins, and lipids, thereby elevating the risk of systemic diseases, including cancer [3,4]. Saliva plays a critical role as the body’s first line of defense against the oxidative stress induced by tobacco, employing its antioxidant system to neutralize these harmful effects [5,6]. This system, which comprises both enzymatic and non-enzymatic antioxidants, helps maintain the balance of redox reactions, safeguarding against disruptions in oral and systemic redox homeostasis [7].

The assessment of total salivary antioxidant levels, using methods such as the ferric reducing antioxidant power (FRAP) assay, offers insights into saliva's antioxidant capacity [8]. While previous studies have explored the impact of tobacco on salivary antioxidants [9,10], there is a gap in research regarding comparisons of salivary antioxidants among smokeless tobacco users with clinical manifestations of tobacco pouch keratosis.

Hence, this study aims to compare the salivary antioxidant systems and oral health parameters such as salivary flow rates and pH levels among smokeless tobacco users with and without clinical manifestations of tobacco pouch keratosis.

## Materials and methods

This clinical cross-sectional comparative study was conducted over a period of two months (January to February 2024), following approval from the Institutional Ethics Committee. Participants were recruited from among patients and their accompanying persons visiting the dental outpatient department. All participants provided written informed consent and met the study’s inclusion criteria.

Sample size determination

The sample size was determined using G*Power software for a one-way ANOVA. Based on a standard effect size of 0.4, a 95% confidence level, 80% power, and values for the standard deviation (σ) and the minimum clinically significant difference (d) obtained from prior studies [11], the required sample size was calculated to be 20 participants per group, totaling 60 subjects.

 Study population

**Figure 1:**
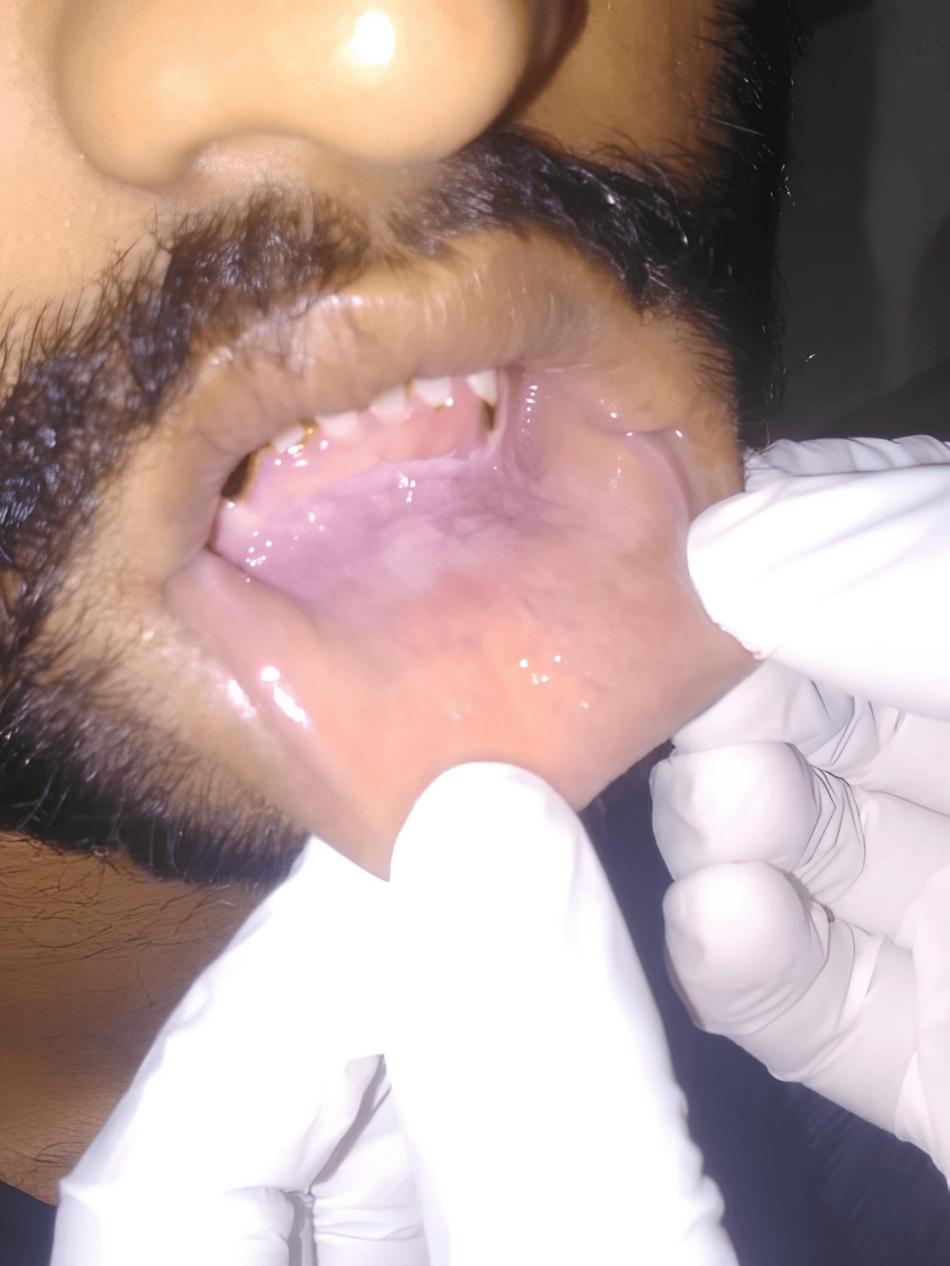
Patient with clinical signs of tobacco pouch keratosis in the mandibular labial vestibule (group 2, case 1)

A total of 63 participants were enrolled in the study and assigned to one of the following three groups: group 1 (n = 23), which included individuals with a history of daily chewable tobacco use for at least five years, without any clinical oral lesions; group 2 (n = 20), which included individuals with the same tobacco use history as group 1, but with clinically evident tobacco pouch keratosis (Figure 1); and group 3 (n = 20), which included healthy control participants, age- and gender-matched, with no history of tobacco use in any form.

Inclusion criteria

The Inclusion criteria were as follows:

Group 1: daily use of chewable tobacco for a minimum of five years; no smoking history.

Group 2: same criteria as group 1, with the presence of clinically diagnosed tobacco pouch keratosis.

Group 3: no history of tobacco use (smoking or chewing) throughout their lifetime.

Exclusion criteria

The study excluded individuals who used both smoked and smokeless forms of tobacco; those with oral mucosal lesions unrelated to tobacco use or with systemic illnesses; individuals with a history of chronic alcohol consumption; and those with a poor Oral Hygiene Index-Simplified (OHI-S, 1964) score or with clinical attachment loss of less than 4 mm.

Study protocol

All participants underwent a comprehensive examination following the completion of a detailed case history form, which included medical and personal history such as dietary habits and oral hygiene practices. For groups 1 and 2, additional data regarding tobacco habits were recorded, including duration of use, type of tobacco (commercial or non-commercial), and quantity consumed per day. In group 2, the location and size of intraoral tobacco pouch keratosis lesions were documented and photographed.

Dental caries status was assessed using the Decayed, Missing, and Filled Teeth index, and oral hygiene status was evaluated using the oral hygiene index-simplified (OHI-S). The study protocol also included the collection of unstimulated saliva samples, which were subsequently analyzed for salivary flow rate, pH level, and total antioxidant capacity.

Unstimulated saliva sample was collected using the spitting method [12]. Participants were instructed to refrain from consuming any food or beverages (except water) for at least one hour before the test session. They were also advised to rinse their mouths several times with deionized (distilled) water and then relax for 5 minutes. Saliva was allowed to accumulate in the mouth, and the participants then spat the saliva into a graduated test tube every 60 seconds for a total of 5 minutes, allowing for the measurement of the salivary flow rate.

The pH of the saliva was measured using a digital three-way pH meter. Between each reading, the electrode was rinsed with distilled water and calibrated using standard pH solutions of 7.0 and 4.0 to ensure stable readings and monitor any drift. The mean of three readings was recorded for each sample.

The saliva sample was then immediately centrifuged at 800 RPM (g) for 10 minutes at 4°C in a digital centrifuge to remove cell debris. The resulting supernatant was promptly stored at -80°C in a Thermo Fisher Scientific deep freezer (Thermo Fisher Scientific, Waltham, MA) for later analysis. All the samples were blinded by assigning a unique non-identifiable code by the research assistant, who was not involved in data collection or analysis. All the samples were prepared in identical containers to prevent bias.

The total antioxidant capacity of all the thawed saliva samples was measured using the FRAP assay. The FRAP reagent (AMD Labs, Peenya, Bangalore, India) was prepared by mixing 25 mL of acetate buffer, 2.5 mL of ferric tripyridyltriazine (TPTZ) solution, and 2.5 mL of FeCl3·6H2O solution. The FRAP assay utilizes the reducing potential of antioxidants to react with a ferric TPTZ complex, producing a colored ferrous TPTZ form. The change in absorbance at 593 nm was measured using a spectrophotometer (UV Digital Spectrophotometer 117, Systronic, Ahmedabad, Gujarat, India) and compared with a standard to determine the antioxidant potential of the sample [11-13]. The sample code keys of blinded saliva samples were revealed after completion of all measurements.

The recorded salivary flow rate, pH, and calculated antioxidant levels were entered into an Excel spreadsheet and analyzed using SPSS Statistics for Windows, Version 22.0 (IBM Corp., Armonk, NY, USA). Categorical variables were summarized as frequencies and percentages, while continuous variables were expressed as means and standard deviations.

In the present study, appropriate statistical tests were selected based on the distribution and nature of the data. Normality testing was performed for all continuous variables using the Shapiro-Wilk test, which guided the selection of either parametric tests (such as one-way ANOVA for normally distributed variables like salivary flow rate and pH) or non-parametric tests (such as the Kruskal-Wallis and Mann-Whitney U tests for variables like antioxidant levels, duration, and frequency of tobacco use). Specifically, the Mann-Whitney U test was employed to compare two independent groups (group 1 vs. group 2) when data were non-normally distributed. Similarly, Spearman’s correlation was used to assess the monotonic relationships between antioxidant levels, tobacco use patterns, and oral health parameters, as it is suitable for non-parametric data and does not assume linearity. This analytical approach ensured statistical robustness and accurate interpretation of the study findings. A p-value of <0.05 was considered statistically significant.

## Results

This study showed varying age distributions across groups, with mean ages of 36.22, 44.10, and 41.10 years for groups 1, 2, and 3, respectively (Table 1). Gender distribution revealed a significant male predominance in groups 1 (82.6%) and 2 (95%) in contrast to group 3 (p < 0.001) (Table 2).

**Table 1:** Age distribution among the study subjects between the three groups ^a^Kruskal-Wallis test

Variable	Category	Group 1		Group 2		Group 3		ꭓ^2 ^-value	p-value
		Mean	SD	Mean	SD	Mean	SD		
Age (years)	Mean	36.22	10.76	44.10	11.39	41.10	13.29	4.055	0.13^a^
	Range	21-55		28-67		22-65			

**Table 2:** Gender distribution among the study subjects between the three groups ^a^Chi-squared test. *Statistically significant

Variable	Category	Group 1		Group 2		Group 3		ꭓ^2 ^-value	p-value
		n	%	n	%	n	%		
Gender	Males	19	82.6%	19	95.0%	8	40.0%	17.045	<0.001*,^a^
	Females	4	17.4%	1	5.0%	12	60.0%		

In group 1, 82.6% of participants used commercial tobacco, while 17.4% preferred non-commercial varieties. In group 2, tobacco consumption was more varied: 50% used commercial tobacco, 45% used non-commercial varieties, and 5% used both types (Table 3).

**Table 3:** Comparison of type of habits between group 1 and group 2 using the chi-squared test

Variable	Category	Group 1		Group 2		ꭓ^2 ^-value	p-value
		n	%	n	%		
Habit	Non-commercial	4	17.4%	9	45.0%	5.534	0.06
	Commercial	19	82.6%	10	50.0%		
	Both	0	0.0%	1	5.0%		

**Figure 2:**
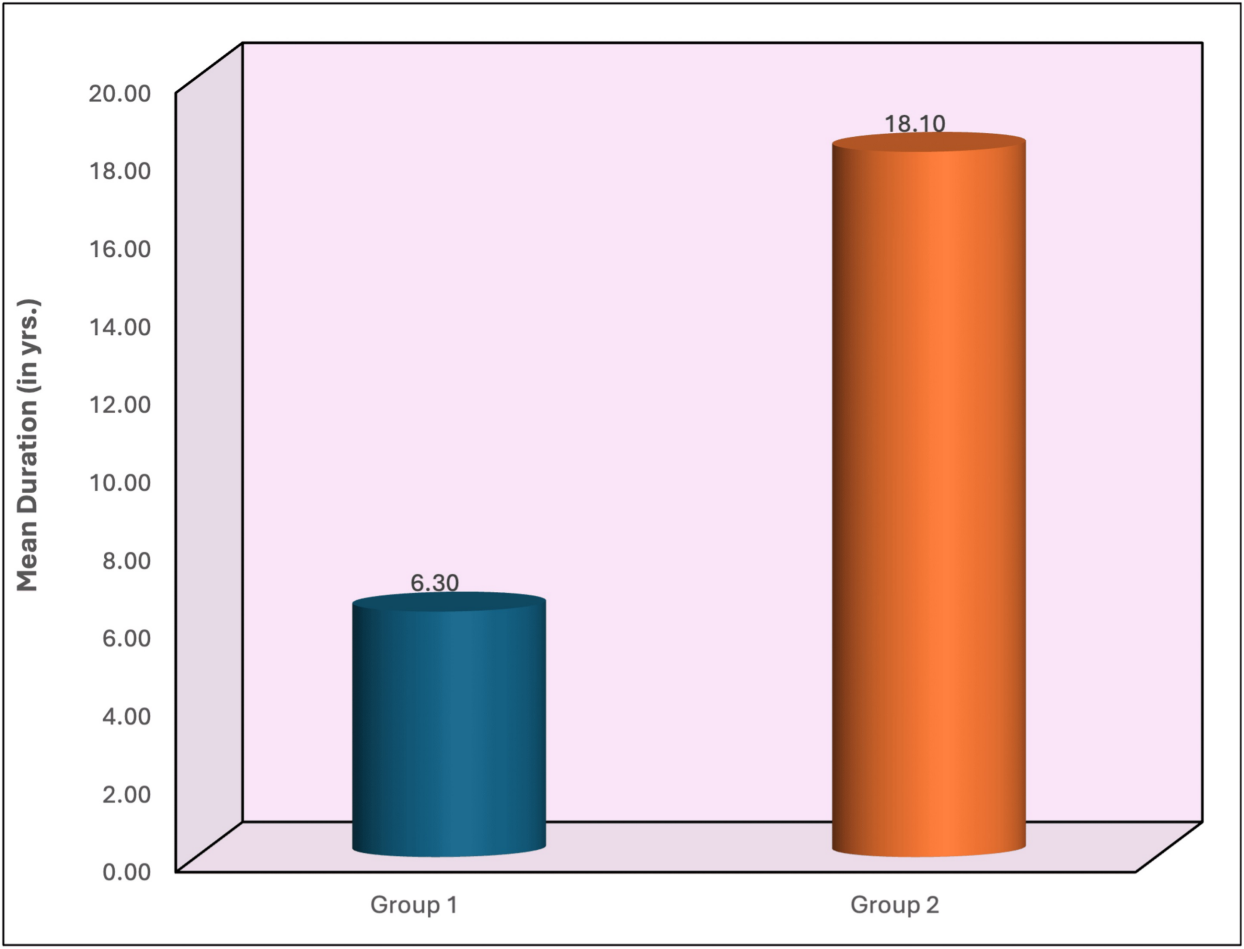
Mean duration of tobacco use (in years) between group 1 and group 2

Figure 2 shows that the average duration of tobacco use was significantly longer in group 2 (18.10 years) than in group 1 (6.30 years), with the Mann-Whitney U test indicating a significant difference (p < 0.001).

**Figure 3:**
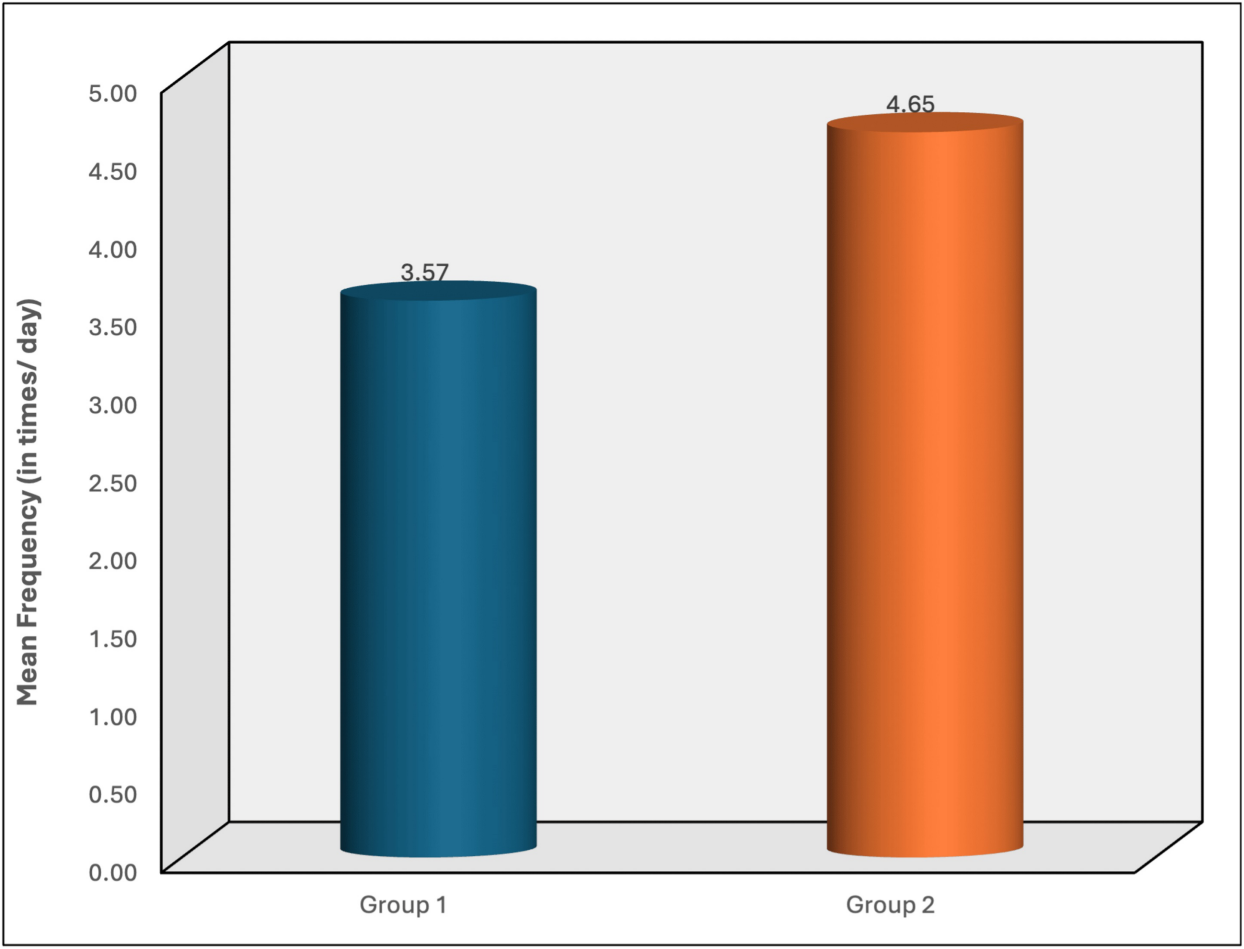
Mean frequency of tobacco use (in times/day) between group 1 and group 2

Figure 3 shows a slightly higher average frequency of tobacco use in group 2 (4.65 times/day) compared to group 1 (3.57 times/day), but this difference was not statistically significant (p = 0.11). These findings highlight distinct tobacco consumption patterns between individuals with and without clinical signs of tobacco pouch keratosis.

One-way ANOVA followed by Tukey's post-hoc test revealed significant differences in mean salivary flow rate and pH levels among the three groups. Group 2 had a significantly lower mean salivary flow rate (0.159 ± 0.094 mL/min) compared to group 1 (0.272 ± 0.137 mL/min) and group 3 (0.277 ± 0.192 mL/min), with p-values of 0.02 and 0.04, respectively. However, no significant difference was observed between group 1 and Group 3. Regarding salivary pH levels, group 2 also exhibited a significantly lower mean pH (6.650 ± 0.355) compared to group 1 (6.999 ± 0.322) and group 3 (6.943 ± 0.360), with p-values of 0.004 and 0.03, respectively. Similarly, no significant difference in mean pH was found between group 1 and group 3 (Table 4).

**Table 4:** Comparison of mean salivary flow rate (mL/min) and salivary pH levels between the three groups using one-way ANOVA followed by Tukey's post-hoc test ^a^One-way ANOVA. ^b^Tukey’s post-hoc test. *Statistically significant G, group

Parameter	Groups	N	Mean	SD	F-value	p-value^a^	Sig. diff	p-value^b^
Saliva flow rate	G1	23	0.272	0.137	4.258	0.02*	G1 vs G2	0.04*
	G2	20	0.159	0.094			G1 vs G3	0.99
	G3	20	0.277	0.192			G2 vs G3	0.04*
Salivary pH	G1	23	6.999	0.322	6.143	0.004*	G1 vs G2	0.004*
	G2	20	6.650	0.355			G1 vs G3	0.85
	G3	20	6.943	0.360			G2 vs G3	0.03*

The comparison of mean dental caries scores and total antioxidant levels among the three groups was conducted using the Kruskal-Wallis test followed by Dunn's post-hoc test. While there were no significant differences in mean dental caries scores across the groups, significant variations were observed in total antioxidant levels. Group 3 (healthy controls) had significantly higher mean total salivary antioxidant levels compared to both group 1 (tobacco users) and group 2 (individuals with tobacco pouch keratosis), with p-values of <0.001. However, no significant difference in total salivary antioxidant levels was found between group 1 and group 2.

Spearman's correlation test revealed a significant relationship between total salivary antioxidant levels and tobacco use parameters across the three groups. In group 1, a moderate negative correlation was observed between total salivary antioxidant levels and the duration of tobacco use, suggesting that longer tobacco use was associated with lower antioxidant levels. A similar strong negative correlation was found in group 2. However, no significant correlations were observed between total salivary antioxidant levels and other parameters in any of the groups (Table 5).

**Table 5:** Spearman's correlation test to determine the relationship between TAL, tobacco habit parameters, and oral parameters *Statistically significant. The correlation coefficients are denoted by 'Rho'. ^a^Denotes negative correlation. Correlation coefficient range: 0.0 indicates no correlation, 0.01-0.20 indicates very weak correlation, 0.21-0.40 indicates weak correlation, and 0.41-0.60 indicates moderate correlation. SFR, salivary flow rate; TAL, total antioxidant levels

Groups	Variable	Values	Duration	Frequency	SFR	pH	Caries
Group 1	TAL	Rho	0.46^a^	0.02^a^	0.28	0.02^a^	0.19
		p-value	0.03*	0.93	0.20	0.93	0.40
Group 2	TAL	Rho	0.63^a^	0.14^a^	0.14^a^	0.05	0.07^a^
		p-value	0.003*	0.56	0.57	0.83	0.77
Group 3	TAL	Rho	-	-	0.06^a^	0.33	0.01^a^
		p-value	-	-	0.81	0.16	0.99

## Discussion

Estimating salivary antioxidant levels shows potential as a biomarker for diagnosing and predicting oral tissue damage and dysplasia, emphasizing tobacco's significant role in the progression toward precancerous and cancerous lesions [14]. Previous studies suggest that discontinuing tobacco use can restore mucosal health in most patients with tobacco pouch keratosis within two to six weeks [3,15,16]. However, if antioxidant levels are depleted, supplementing with antioxidants may accelerate regression, potentially offering an early intervention to prevent dysplastic changes in the oral cavity [17-19]. The findings of this study underscore the complex relationship between smokeless tobacco use, oral health, and salivary antioxidant systems.

The observed variability in age distribution across the study groups in the present study highlights the diverse demographic profile of tobacco users. Notably, individuals who exhibited clinical signs of tobacco pouch keratosis, were generally older, with a mean age higher than that of individuals, who used chewable tobacco but did not have keratosis. This age disparity likely reflects prolonged and escalating tobacco exposure in those with tobacco pouch keratosis, emphasizing the cumulative effects of long-term tobacco use on oral health.

In a 2024 study by Nautiyal et al., which included 5,613 patients with various oral lesions, the average age was 37.28 years. The most common lesion observed was frictional keratosis (4,026 patients), followed by tobacco pouch keratosis (1,537 patients) and morsicatio buccarum (54 patients), with all lesions being more prevalent in men [20]. The gender distribution patterns in the present study further revealed a significant male predominance among individuals with established tobacco chewing habits and those with tobacco pouch keratosis, consistent with existing epidemiological data that report higher tobacco consumption rates among men [21].

Examining tobacco usage patterns reveals subtle differences between individuals with and without clinical signs of tobacco pouch keratosis. While both groups primarily use commercial tobacco products, those with tobacco pouch keratosis tend to have more prolonged and frequent tobacco use, as evidenced in the present study. These findings underscore the progressive nature of tobacco-induced changes in the oral mucosa, where extended exposure correlates with more severe clinical manifestations [22]. Previous research consistently shows that prolonged and frequent tobacco use contributes to various oral health issues, including mucosal lesions and potentially malignant disorders [23].

By understanding tobacco usage patterns and promoting early intervention strategies, healthcare providers can play a crucial role in mitigating the impact of tobacco-related oral changes and improving overall oral health outcomes [24,25].

Saliva plays a crucial role in defending the body against oxidative stress, particularly from tobacco exposure, due to its robust antioxidant system [26]. However, chronic tobacco use can disrupt the balance of antioxidants in saliva, impairing its protective function. Assessing salivary parameters such as flow rate, pH, and total antioxidant capacity can provide valuable insights into the effects of tobacco on oral health [9,17,26].

The FRAP assay is one of the most widely used methods to investigate total antioxidant levels of various biological fluids. FRAP assay is a simple, fast, and cost-effective method for the assessment of salivary antioxidant capacity and can help clarify the extent of oxidative stress-induced damage in tobacco users [8].

In the present study, individuals with tobacco pouch keratosis exhibited significantly lower salivary flow rates and pH levels compared to both tobacco chewers without pouch keratosis and healthy individuals without habits. These findings align with previous research suggesting that smokeless tobacco use can disrupt salivary function, leading to reduced saliva production and altered pH levels [26]. Differences in salivary flow rates and pH levels between tobacco users and healthy individuals in this study suggest that tobacco exposure alters saliva composition.

Lower salivary flow rates can compromise the protective functions of saliva, such as buffering acids and clearing food debris and bacteria, which may increase the risk of oral diseases. Similarly, a decrease in salivary pH could lead to a more acidic oral environment, promoting the development of dental caries and other oral health problems [26,27].

In this study, no significant differences were found in mean dental caries scores across groups, but notable variations in total salivary antioxidant levels were observed. Healthy controls had significantly higher antioxidant levels than tobacco users and individuals with tobacco pouch keratosis, highlighting the impact of tobacco use on saliva's antioxidant capacity as previously reported [11]. Furthermore, these findings align with those reported by Chauhan et al., suggesting that tobacco use reduces antioxidant levels and increases oxidative stress in the oral cavity [17]. Antioxidant levels may initially increase as a defense mechanism, but with prolonged use, these defenses weaken, resulting in a reduction of superoxide dismutase and the development of oral lesions [17,26]. Ahmad et al. further emphasize that pro-oxidant changes in smokeless tobacco users, such as those using naswar, contribute to negative health outcomes, including oral cancer [28].

Limitations

Participant selection was carefully conducted to minimize bias related to diet, nutrition, systemic diseases, and oral hygiene. However, the study has certain limitations. One notable limitation is the reliance on self-reported data regarding the frequency, duration, and onset of tobacco pouch keratosis, which may be subject to recall bias. Additionally, the FRAP assay presents methodological limitations, including its inability to detect antioxidants containing thiol groups and its dependence on the Fe³⁺/Fe²⁺ redox potential. Compounds with a redox potential lower than that of Fe³⁺/Fe²⁺ may lead to falsely elevated results, and substances such as uric acid in saliva can interfere with the accuracy of the FRAP assay. Future prospective studies are warranted to quantify total salivary antioxidants more precisely, correlate findings with clinicopathological features, and evaluate the impact of antioxidant-based interventions.

## Conclusions

This study highlights the harmful effects of smokeless tobacco on oral health, specifically its impact on salivary flow rates, pH, and antioxidant capacity. It suggests mechanisms that contribute to tobacco-induced changes in the oral mucosa, emphasizing the need for interventions to mitigate these effects. The findings underscore the importance of tobacco cessation programs and early detection of oral lesions.

In conclusion, the study stresses the need for comprehensive oral health assessments in tobacco users, particularly those with tobacco pouch keratosis. Future research should focus on longitudinal studies to track changes in salivary parameters and antioxidant levels, providing deeper insights into tobacco-related oral damage. Addressing the effects of smokeless tobacco requires a multifaceted approach, including public health initiatives, targeted interventions, and ongoing research.
